# Aptamers, the Nucleic Acid Antibodies, in Cancer Therapy

**DOI:** 10.3390/ijms21082793

**Published:** 2020-04-17

**Authors:** Zhaoying Fu, Jim Xiang

**Affiliations:** 1 Department of Biochemistry and Molecular Biology, College of Medicine, Yanan University, Yanan 716000, China; 2 Division of Oncology, University of Saskatchewan, Saskatoon, SA S7N 4H4, Canada

**Keywords:** aptamer, SELEX, cancer, therapy

## Abstract

The arrival of the monoclonal antibody (mAb) technology in the 1970s brought with it the hope of conquering cancers to the medical community. However, mAbs, on the whole, did not achieve the expected wonder in cancer therapy although they do have demonstrated successfulness in the treatment of a few types of cancers. In 1990, another technology of making biomolecules capable of specific binding appeared. This technique, systematic evolution of ligands by exponential enrichment (SELEX), can make aptamers, single-stranded DNAs or RNAs that bind targets with high specificity and affinity. Aptamers have some advantages over mAbs in therapeutic uses particularly because they have little or no immunogenicity, which means the feasibility of repeated use and fewer side effects. In this review, the general properties of the aptamer, the advantages and limitations of aptamers, the principle and procedure of aptamer production with SELEX, particularly the undergoing studies in aptamers for cancer therapy, and selected anticancer aptamers that have entered clinical trials or are under active investigations are summarized.

## 1. Introduction

The advent of the technology of using B lymphocyte hybridomas to produce monoclonal antibodies (mAbs) in the 1970s gave the medical world a ray of hope to conquer cancers because mAbs are capable of binding to target molecules, such as tumor antigens specifically, and carrying cytotoxic agents selectively to cancer cells, which means effective destruction of cancer cells with minimum damage to normal cells. In the years that followed, mAbs demonstrated their exceptional usefulness particularly in biomedical researches and also in clinical diagnoses and treatment of a few types of cancers; nevertheless, because of a number of reasons, mAbs did not achieve the expected wonder in cancer therapy on the whole.

The 1990s of the last century witnessed another technology of making biomolecules capable of specific binding. The technique was dubbed systematic evolution of ligands by exponential enrichment (SELEX) and the molecules it produced were named aptamers [1,2]. Aptamers are single-stranded nucleic acids (either DNA or RNA) that can recognize targets through three-dimensional complementarity (rather than base pairing) and bind to targets with high specificity and affinity; hence, they have been described as nucleic acid antibodies [3]. Similarly to mAbs, aptamers can be used in laboratory research and in clinical diagnosis and treatment, but aptamers have some advantages over mAbs particularly in in vivo applications, one of which is that they have little or no immunogenicity and thus do not elicit immunological rejections, meaning the feasibility of repeated use and also fewer side effects [4]. There has already been an aptamer called Macugen (Pegaptanib Sodium Injection) being approved by the US Food and Drug Administration (FDA) for therapeutic application. This aptamer targets vascular endothelial growth factor (VEGF) and is used for the treatment of age-related macular degeneration (AMD) [5].

This paper summarizes the general properties of the aptamer, the advantages and limitations of aptamers compared with mAbs, the principle and procedure of aptamer production with SELEX, (particularly) the undergoing investigations into aptamer’s applications in cancer treatment, and selected aptamers that have entered clinical trials or are under active investigation for cancer therapy.

## 2. General Properties of the Aptamer

Single-stranded nucleic acid molecules can bind to complementary sequences via base pairings, but they can also form multiple secondary structures (such as stem-loops, hairpins, bugle, pseudoknots, etc.) and further form unique three-dimensional structures. Thus, they can bind to a great variety of targets including proteins, cells, bacteria, virions, and various small organic and inorganic molecules through closely matched spatial complementarity and by means of electrostatic/ionic interaction, hydrogen bonding, van der Waals’ force, as well as hydrophobic interactions [6]. Exploiting the latter properties, Gold lab and Szostak lab of the United States in the early 1990s independently established a kind of in vitro evolution technique that can generate oligonucleotides with high specificity and binding affinity [1,2]. The Szostak group gave the technique the name “in vitro selection” while the Gold group named it systematic evolution of ligands by exponential enrichment or SELEX; later literature all adopted the latter term. The Szostak group first called the molecule aptamer and the name has been accepted universally. Aptamers are single-stranded DNA (ssDNA) or single-stranded RNA (ssRNA) oligonucleotides of variable lengths (usually 20–80 nt residues). The sequence in the central region of the molecules, generated during chemical synthesis and selection, is highly random and can bind to various targets, while the flanking sequences, predesigned by the researcher, are constant and are used for aptamer selection or production (the sequences on both sides can be truncated or trimmed after the final selection). Aptamers can form diverse three-dimensional structures and can thus bind to a variety of targets specifically and tightly. The binding affinities (dissociation constants) of aptamers are usually in the range of low nanomolar to high picomolar but may reach femtomolar [7,8]. The recognition is highly exquisite and can discriminate structural differences between enantiomers (mirror images with identical chemical composition) and the presence or absence of a hydroxyl group [9].

Even though aptamers are highly specific, the issue of off-target binding must still be carefully investigated for them to meet regulations and enter clinical use. To date, quite a few aptamers have been shown to have no or negligible target toxicity (not to bind to off-target cells/tissue) [10,11,12,13,14,15,16,17].

## 3. Advantages and Limitations of Aptamers

Aptamers can bind to targets with high specificity and affinity, similar to mAbs, and they are also similar to mAbs in terms of biomedical applications. However, aptamers have a significant advantage over mAbs in medications because they have no or little immunogenicity and will not cause immune rejection. Experiments using monkeys demonstrated that there was no or only negligible immune response even when the dose was 1000 times higher than the normal therapeutic dose [18,19]. Moreover, aptamers can conjugate with and deliver various therapeutics into cells in addition to affecting target molecules directly [20]. Refer to Table 1 for a comparison between aptamers and mAbs.

Aptamers have other advantages as well: (1) Aptamers have a broader spectrum of targets than mAbs—they can be raised against non-immunogenic substances such as small organic and inorganic molecules and even metal ions; they can be made against strong bio-toxins because they do not need animal immunization for production [25]. (2) The manufacture of aptamers is less time consuming because they are made in vitro thus avoiding the long time animal immunization; in addition, the affinity optimization and manufacturability improvement for the production of mAbs may require even more time. [20]. (3) Aptamers can easily be modified to increase their applications and stability. Stability is especially important for RNA aptamers because RNA aptamers are liable to degradation by nucleases when used in vivo [26]. (4) Aptamers have less batch-to-batch variations [27,28,29,30]. (5) The cost of making aptamers is lower than that of making mAbs [24]. (6) Aptamers can be cloned into vectors and stored for a very long time, and can be produced any time in need by bacterial expression and/or PCR amplification. Alternatively, after the sequence of aptamers have been obtained, they can be chemically synthesized and PCR amplified at any time. (7) Aptamers are not sensitive to temperature: they can tolerate 80 °C while mAbs will generally denature at this temperature. Even if they are denatured, aptamers can easily be renatured to their original three-dimensional structure [27]. (8) Aptamers have a smaller molecular weight (~20,000 Da vs. ~150,000 Da for mAbs) and are easy to penetrate tissues such as tumors when used for therapeutic purposes [27].

The main limitations of aptamers lie in their susceptibility to degradation by nucleases (this can be overcome by aptamer modification and is discussed in Section 4.3 of the text), the difficulty to select aptamers against some targets, and the rapid renal clearance [31]. In theory, aptamers can be produced against any type of targets, from small inorganic and organic molecules to cells and to whole organisms such parasites, but in practical situations, it is sometimes difficult to obtain high-specific and high-affinity aptamers to some targets [32]. For instance, it is difficult to select aptamers against negatively charged targets because oligonucleotide strands have negative charges on their backbone [33]. The low molecular weight of aptamers is both advantageous and disadvantageous in terms of in vivo application. The small size makes them easier to penetrate tissue, an advantage for therapy as well as in vivo imaging; it also makes them faster to be cleared from the kidney, which means shorter half-life and is disadvantageous for therapy but advantageous for in vivo imaging [34]. Although the disadvantage of short circulating half-life can be overcome by conjugating aptamers with an inert molecule such as polyethylene glycol or cholesterol, this may meanwhile retard tissue uptake; hence, there is a paradox and the designer of aptamers should take all these into consideration and make a good balance between them [26,35,36,37].

## 4. Principle and Procedure of SELEX

**Figure 1:**
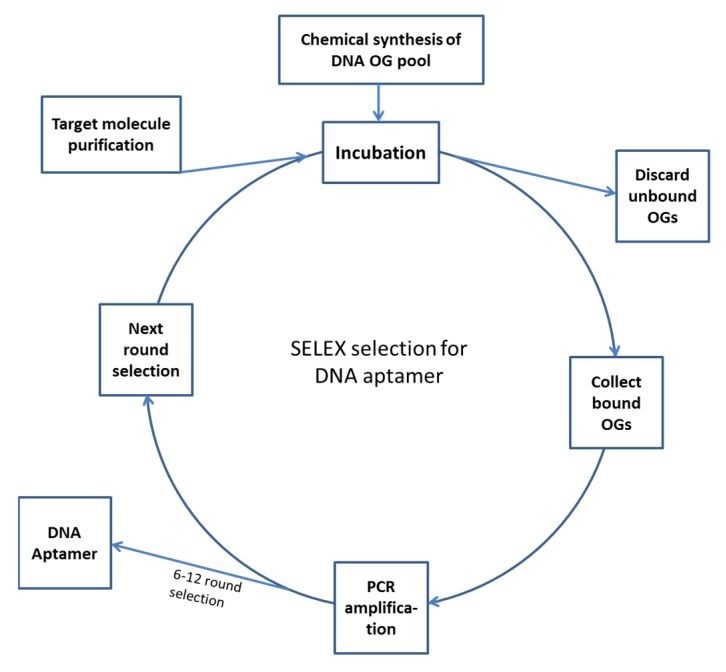
Selection of DNA aptamer using SELEX. Note: For the selection of RNA aptamer, the DNA oligonucleotide pool must be in vitro transcribed into RNA oligonucleotide pool before selection, and the collected oligonucleotides must be reverse transcribe-amplified with TR-PCR into DNA and then be in vitro transcribed into RNA for the next round of selection. Abbreviations: OG, oligonucleotide; PCR, polymerase chain reaction, SELEX, systematic evolution of ligands by exponential enrichment.

The principle of aptamer production using SELEX includes first the chemical synthesis of a DNA pool containing about 10^14–17^ different oligonucleotide molecules. Because the pool is huge, there must be some chains that can bind to a specific target with high affinity. The task of the ensuing work is to screen out and amplify these specific oligonucleotides, which usually requires several cycles of selection and amplification. With the progression of the selection process and the increased stringency of selection conditions, the affinity of the selected oligonucleotides to the target increases gradually. In addition, because the PCR amplifications are not accurate (this may be done intentionally by using low fidelity Taq polymerase), “point mutations” may take place, which results in the generation of new oligonucleotide chains, and the binding affinity and specificity of the oligonucleotides may improve or be enriched in the course of selection. The finally resulting DNA or RNA oligonucleotides that bind to the target very tightly and specifically are the desired aptamers for the target [29,38,39,40]. The typical procedure of SELEX includes the following steps (see Figure 1).

### 4.1. Preparation of DNA Oligonucleotide Pool

Whether you are to make DNA aptamers or RNA aptamers, the initial step is the preparation of a DNA oligonucleotide pool by chemical synthesis. While the random central sequences of the oligonucleotides are generated during the random chemical synthesis, the constant sequences on both ends must be predesigned. The constant regions contain primer binding sequences for PCR amplification and are commonly 20–30 nucleotides in length. The constant regions may also include restriction sites for aptamer cloning. If RNA aptamers are to be made, the binding site for RNA polymerase (such as bacteriophage T7 promoter) must also be included in a constant region [41]. 

The length of the central region should be considered carefully in designing an oligonucleotide pool. Shorter chains are easier to control chemically, however, if the central region is too short, it cannot form enough secondary structures to bind to the target stably and specifically, and there may not be enough kinds of three-dimensional structures in the pool for screening (that is, the sequence space of the pool is not large enough). In theory, the kinds of oligonucleotide chains that can be formed in the random oligonucleotide pool are four to the power of *n* (4^n^), and *n* is the base number of the random nucleotides in the central region. For example, an oligonucleotide pool can have approximately 10^15^ different chains if the central region has 25 nucleotides (4^25^ ≈ 10^15^) [41].

Generally, the sequence space of the oligonucleotide pool increases with the length of the central region (i.e., the available three-dimensional structures in the pool increase with central region length). However, when the length reaches a certain degree, the kinds of three-dimensional structures that can be formed do not increase significantly with the increase of length. For example, the types of oligonucleotide chains that can be formed in the oligonucleotide pool containing 30 random nucleotides are only about 1/1000 of the calculated number. Therefore, the central random sequence is typically designed to be 24–40 nucleotides long, which can already form diverse enough three-dimensional conformations to bine almost all kinds of target molecules in nature [41]. Once the sequence has been designed with satisfaction, the oligonucleotide pool can be synthesized by a DNA synthesizer or by a commercial company.

### 4.2. Selection and Enrichment of Aptamers

After the oligonucleotide pool has been chemically synthesized, it must be amplified using PCR and then converted to ssDNA oligonucleotides before any selection begins. Several selection methods based on SELEX are available at present; these include affinity chromatography, nitrocellulose membrane filtration, magnetic bead separation, capillary electrophoresis, microfluidic selection, microarray method, etc. Besides, semi-automatic or automated SELEX screening systems have also been established [42]. 

The screening and enrichment program of DNA aptamers begins with incubating the single-stranded oligonucleotide pool with target molecules or cells under proper selection conditions; then, the unbound or loosely bound oligonucleotides are washed out. Next, the bound oligonucleotides are separated from target molecules and collected, and the collected oligonucleotides are PCR amplified, which completes the first round of selection. The PCR product is then used to carry out the second round of selection, and so on. Generally, 6–14 cycles of screening and enrichment are required to obtain the desired aptamer. For the production of RNA aptamers, the initial DNA oligonucleotide pool must be in vitro transcribed into an RNA oligonucleotide pool before screening; the selected RNA oligonucleotides must be reverse transcribed into DNA by RT–PCR (the number of the molecules are amplified in the course) and then be in vitro transcribed into RNA molecules for the next round of screening and enrichment. Owing to the low fidelity of DNA polymerase used in PCR, some variants will be introduced in each PCR cycle; as a result, the binding capacity of oligonucleotide pool gradually increases in the screening and amplification process [43,44]. 

A counter selection or negative selection is usually necessary before the SELEX selection, whether it is for DNA aptamer or RNA aptamer production. The purpose of the counter selection is to remove any oligonucleotides that will bind to the immobilizer, the matrix/material used for immobilization of the target molecules, such as the magnetic beads or nitrocellulose membrane. In a counter selection, the DNA or RNA pool is first incubated together with the supporting matrix/immobilizing material and then the bound oligonucleotides are discarded and the unbound oligonucleotides are collected and used for the (positive) SELEX selection [45,46].

### 4.3. Aptamer Sequencing, Characterization, and Modification

When aptamers have been successfully selected, they should be cloned into vectors, their base sequence determined, and their possible secondary structure, target-binding affinity, stability, and some other characteristics analyzed [47]. 

Therapeutic aptamers, particularly RNA aptamers, frequently require modifications because they are sensitive to nucleases and are easily degraded in vivo, which shortens their half-life and limits their applications. Modification methods include: (1) Using chemically modified bases—this is done by adding nucleotide triphosphates with chemically modified bases (such as five-position modified uridine) to the reaction system [48]. (2) Modifying the phosphate–sugar backbone—this is usually done by adding 2′-modified nucleotide triphosphates (such as the 2′-position modification of fluorine, methoxy, or amino group) to the reaction system [49,50,51]. The above modifications depend on the ability of RNA polymerase to incorporate modified nucleotide triphosphates to the growing chain. (3) Conjugating high molecular mass moieties such as polyethylene glycol or fatty acid to the 5′ end or the 3′ end of the RNA aptamer [52,53]. (4) Producing mirror-image aptamers—this process involves creating a chemical mirror image (an enantiomer or optical isomer) of the target molecule, selecting aptamers against this mirror image, and, finally, creating (chemically synthesizing) a mirror image of the aptamer. These types of aptamers are called Spiegelmers (Spiegel is the German word for mirror) [54,55,56,57,58]. The above modifications can all increase the half-life of the aptamers by making them insensitive to nucleases, whereas the third method, conjugating high molecular mass moiety, can, meanwhile, increase the renal retention time of the molecules; the third method can be conducted in combination with other modifications [59].

## 5. Aptamers in Cancer Therapy

Taking together all of the aforementioned advantages, it could be seen that aptamers have promising therapeutic potential in cancer treatment. Aptamers can be used in cancer therapy either by directly inhibiting the activities of target molecules through binding to the targets such as growth factors or oncoproteins, or through guiding and delivering anticancer agents such as chemotherapeutics or siRNAs into cancer cells; aptamers can also block or stimulate immune receptors in lymphocytes to relieve immune suppression or boost immune response against cancer [60,61,62].

### 5.1. Targeted Inhibition

Aptamers can bind to ligands such as growth factors and chemokines and interrupt their interaction with cell receptors, or alternatively, they can bind to cell receptors to block ligand binding [63,64]. When transported into cells, they can also interfere with the functions of cytoplasmic targets [65]. 

#### 5.1.1. Targeting Platelet-Derived Growth Factor (PDGF)

Platelet-derived growth factor (PDGF) regulates blood vessel formation (angiogenesis), mesenchymal cell mitogenesis, as well as chemotaxis. Overexpression of PDGF has been linked to malignancies. PDGFs function as disulfide-linked homodimers PDGF-AA, PDGF-BB, PDGF-CC, PDGF-DD, or heterodimer PDGF-AB. Their receptors are PDGFR-α and PDGFR-β (PDGF-A, PDGF-B, and PDGF-C bind PDGFR-α, whereas PDGF-B and PDGF-D bind PDGFR-β). Upon PDGF binding, the two receptor isoforms dimerize into three possible combinations: αα, ββ, or αβ. PDGFRs are classified as receptor tyrosine kinase; following PDGF activation, they are switched on by auto-phosphorylation of tyrosine residues on their intracellular tyrosine kinase domain [66]. 

Angiogenesis is necessary for tumor growth and is a prerequisite for cancer metastasis via blood vessels. Inhibition of angiogenesis has been considered as one of the promising measures to treat cancers. DNA aptamers against PDGFs were first developed by Green et al. [67]. These oligonucleotides bind to PDGF-AB and PDGF-BB with much higher affinity (Kd ≈ 10^−10^ M) than to PDGF-AA (Kd > 10^−8^ M), which indicates that the aptamers have a specific recognition of the PDGF-B-chain in the context of PDGF-AB heterodimer or PDGF-BB homodimer. The aptamers were truncated to determine the minimal sequence length necessary for high-affinity binding. Representative minimal oligonucleotides were shown to be able to inhibit the binding of PDGF-BB but not of PDGF-AA to PDGF receptors PDGFR-α or PDGFR-β in porcine aortic endothelial cells. These aptamers also potently inhibited PDGF-BB-dependent [^3^H] thymidine incorporation in the porcine aortic endothelial cells expressing the PDGFR-β receptor. Therefore, the PDGF aptamer has high promise to be used as an effective and specific inhibitor of PDGF in cancer therapy. Overexpression of PDGF-BB is associated with the development of colorectal cancer. Sae-Lim et al. [68] recently used the above-mentioned PDGF aptamer in their study to inhibit the proliferation of colorectal cancer cells. The results showed that cells treated with the aptamer proliferated slower than control. Western blot revealed a reduced phosphorylation level of ERK1/2 (a key component in the PDGF signaling pathway). It was concluded that the DNA aptamer against PDGF-BB blocked the binding of PDGF-BB to its receptor and inhibited colorectal cancer cell proliferation partly through downregulating the Ras/Raf/MEK/ERK signaling pathway.

Sennino et al. [69] examined the effects of the DNA aptamer AX102, a modified version of the aforementioned aptamer that binds PDGF-B chain selectively, on tumor vasculature. Treatment with AX102 caused the progressive loss of pericytes; reduction of pericytes and endothelial cells further led to empty basement membrane sleeves that gradually became invisible. The end result was inhibited tumor angiogenesis. The magnitude of tumor vessel regression caused by PDGF-B blockade was tumor-specific. Lu et al. [70] tested the efficacy of AX102 aptamer in pericyte regulation in an ovarian cancer model, with or without targeting endothelial cells by Bevacizumab (a VEGF-A neutralizing mAb). The results indicated that dual targeting of pericytes and endothelial cells achieved a much better anti-angiogenesis effect in ovarian carcinoma, resulting in a 76%–88% inhibition of tumor growth. Lately, Falcon et al. [71] found that inhibition of PDGF-B signaling in Lewis lung carcinomas by the DNA aptamer AX102 enhanced the transport and effectiveness of the chemotherapeutic drug cyclophosphamide by augmenting the efficiency of tumor blood vessels; combined application of cyclophosphamide and AX102 also showed synergistic effects on tumor cell proliferation in RIP-Tag2 tumors.

#### 5.1.2. Targeting PDGFR

Platelet-derived growth factor receptor-β (PDGFR-β) is a transmembrane receptor tyrosine kinase. Overexpression of PDGFR-β in endothelial and tumor-associated stromal cells occurs in different human cancers. PDGFR-β expression in certain cancers has been linked with aggressiveness, resistance to therapy and recurrence [72]. Camorani et al. [73] reported that a PDGFRβ-specific RNA aptamer named Gint4.T could specifically bind to the human PDGFRβ ectodomain and cause strong inhibition of receptor activation and of downstream signaling both in continuous cell lines and in primary cultures of human glioblastoma. In addition, the Gint4.T aptamer could significantly inhibit cell migration and proliferation, induce cell differentiation, and impede tumor growth in vivo.

Tumors exhibiting interstitial hypertension have reduced uptake of chemotherapeutics, and PDGFR signaling has been shown to mediate interstitial hypertension. Pietras et al. [74] reported that inhibition of PDGFR signaling by combined treatment with PDGF-B inhibitory aptamers and PDGFR tyrosine kinase inhibitor lowered tumor interstitial hypertension and enhanced drug uptake by the tumor cells. 

Triple-negative breast cancers (TNBCs) do not express estrogen receptor, progesterone receptor, and HER2; these cancers are difficult to treat because of their lack of targetable molecules. Cell-SELEX was exploited to find alternative targets and PDGFR has been suggested as one of them [75]. It has been suggested that PDGFR-β may mark breast cancer cells with stem-like characteristics and/or epithelial-mesenchymal transition. Camorani et al. [76] found the expression of PDGFR-β in a subgroup of mesenchymal tumors with invasive and stem-like phenotype, and suggested a role of PDGFR-β in driving TNBC invasiveness and metastases. They showed that PDGFR-β aptamers inhibited the invasive growth of TNBC cells in three-dimensional culture and blocked migration and invasion of mesenchymal TNBC cells and prevented lung metastases of TNBC cells.

In a separate study, the above research group demonstrated that bone marrow-derived mesenchymal stem cells (BM-MSCs) increased the aggressiveness of TNBC cells as indicated by their ability of migration, invasion, and acquisition of stemness markers. They also showed that treatment of the BM-MSCs with an RNA aptamer against PDGFR-β led to the inhibition of receptor-dependent signaling pathways and thus significantly blocked BM-MSC recruitment towards TNBC cells as well as BM-MSC’s trans-differentiation into carcinoma-associated fibroblast-like cells [77].

#### 5.1.3. Targeting Chemokine

C-X-C chemokine ligand 12 (CXCL12), also known as stromal cell-derived factor 1 (SDF1), is a CXC subfamily chemokine and is the ligand for chemokine receptors CXCR4 and CXCR7. CXCL12 is widely expressed in many tissues and cell types and is strongly chemotactic for lymphocytes. During embryogenesis, CXCL12 directs the migration of hematopoietic cells from fetal liver to bone marrow and is responsible for large blood vessel formation. In adulthood, CXCL12 plays an essential role in angiogenesis by recruiting endothelial progenitor cells from the bone marrow through activating CXCR4 [78]. CXCL12 signaling has also been observed in CXCR4 expressing cancers and is involved in cancer cell invasion and metastasis [79]. The blockade of the CXCL12/CXCR4 passage has, therefore, emerged as a potential way of targeted cancer therapy [80].

In chronic lymphocytic leukemia (CLL), the homing and retention of cancer cells into the lymph nodes and bone marrow is critical to disease development. CLL cells are attracted to the bone marrow through activation of CXCR4 expressed on CLL cells by CXCL12 secreted by stromal cells. CLL cells localized in the bone marrow are protected from anticancer drugs; this can result in the persistence of the residual disease after conventional treatment and favor cancer recurrence [81]. 

NOX-A12 is a mirror-image RNA aptamer, an L-RNA oligonucleotide or the mirror image of naturally occurring D-RNA molecule (Spiegelmer), developed by the biotechnology company Noxxon Pharma in Berlin, Germany [82]. NOX-A12 aptamer is a CXCL12 antagonist that binds to the chemokine and disrupts the homing and the accumulation of CLL cells in the bone marrow, sensitizing these cells to cytotoxic drugs [81]. NOX-A12 molecules are highly resistant to degradation by nucleases owing to their L-configuration. Hoellenriegel et al. [83] inspected the effect of NOX-A12 on CXCL12-induced CLL cell migration and drug resistance; in the experiment, CLL cells were allowed to migrate toward CXCL12 concentration gradient (a chemotactic response) in the absence or presence of NOXA12. They found that NOX-A12 significantly inhibited CXCL12-mediated migration of CLL cells at a very low concentration of 3 nM. They also demonstrated that CXCL12-mediated chemotaxis of two other lymphoid cell lines (Jurkat and Nalm-6) could be reduced effectively in the presence of NOX-A12.

#### 5.1.4. Targeting HER2

Several aptamers targeting human epidermal growth factor receptor 2 (HER2) have been developed but most of them were used to deliver anticancer therapeutics such as cytotoxic agents, therapeutic RNAs, and nanoparticles into cancer cells instead of inhibiting HER2 function directly and so will not be discussed here; readers can refer to the relevant sections and to Table 2.

### 5.2. Targeted Delivery

**Figure 2:**
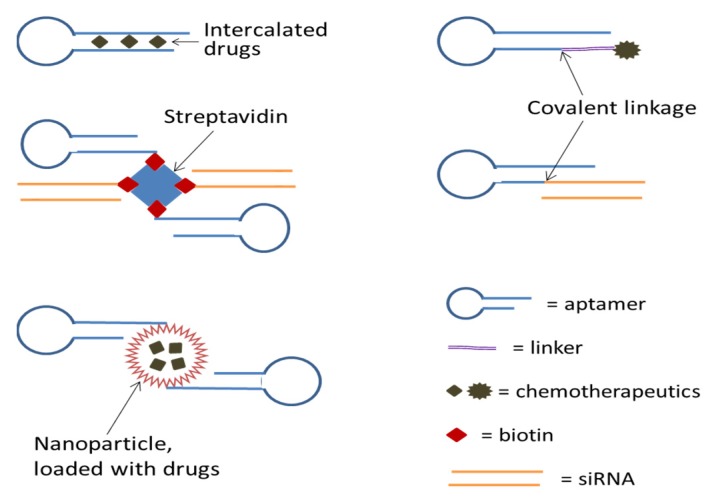
Conjugation of aptamers with anticancer therapeutics for targeted delivery.

Not only can aptamers be selected to target and inhibit cancer-specific molecules, but they can also be selected to recognize the surface structures of specific cell types and some of them can be endocytosed or taken in by the cells. The latter properties can be employed to deliver therapeutic agents such as cytotoxic drugs and therapeutic RNAs selectively into cancer cells to exert their activities while avoiding damaging normal cells [114,115,116,117]. Aptamers used for drug delivery mostly target cell membrane receptors that can internalize upon ligand binding, and successful deliveries of a variety of cargoes targeting different receptors have been reported to date [118] (see Figure 2).

#### 5.2.1. Delivery of Cytotoxic Agents

Chemotherapeutics or other cytotoxic or anticancer drugs can be linked either covalently or non-covalently to cell internalizing aptamers and be transported into cancer cells. Human prostate-specific membrane antigen (PSMA) is a class II membrane glycoprotein that resides mainly in the extracellular space and is highly expressed in normal prostate tissue and prostate cancers; PSMA has an internalizing signal that allows internalization of the bound ligands into the cell [119]. Two PSMA-targeting aptamers, called xPSM-A9 and xPSM-A10 (A9 and A10), were first developed by Lupold et al. [91] early in 2002; they share no consensus sequences and each binds to a unique extracellular epitope of PSMA. The aptamers were subjected to 3′-truncation in their central random regions to identify the minimum required binding elements. One of the aptamers, A10, when the central region was truncated from 40 nt to 25 nt, could still bind to the PSMA-positive LNCaP prostate cancer cells specifically but not to the PSMA-negative PC-3 prostate cancer cells [91]. 

Bagalkot et al. [120] investigated the targeted delivery of the chemotherapeutic drug doxorubicin (Dox) to cancer cells by using the PSMA aptamer A10. Multiple Dox molecules were non-covalently loaded (intercalated) to the double-stranded region of the aptamer A10, and when used in cell culture, resulted in the uptake of the aptamer–Dox conjugates by PSMA-expressing prostate cancer cells and the intracellular release of the Dox molecules. 

Dox has also been covalently linked to a DNA aptamer named sgc8c; the drug was conjugated with the aptamer by a hydrazone linker which allows the release of Dox at pH 4.5–5.5. The Dox-sgc8c conjugate could be efficiently internalized by T-cell acute lymphoblastic leukemia (TALL) cells through binding to protein tyrosine kinase 7, a transmembrane receptor highly expressed on TALL cells, and after internalization, Dox could be cleaved from the conjugate in the acidic endosomal environment because the linkage between Dox and the aptamer had been designed to be acid-labile [121]. 

An aptamer developed against HER2 (the aptamer’s name being HB5) has been used to deliver Dox to breast cancer cells in vitro. The aptamer–Dox complex, which was formulated by intercalating Dox molecules into the aptamers, could selectively deliver Dox to HER2-positive breast cancer cells and retained the cytotoxicity of Dox against these cells; the aptamers preferentially target to HER2-positive breast cancer cells but not to HER2-negative cells [108].

#### 5.2.2. Delivery of Therapeutic RNAs

Cell-specific internalizing aptamers have been used to deliver therapeutic oligonucleotides such as siRNAs, miRNAs, gRNAs, antisense RNAs, as well as protein-binding aptamers into cancer cells. Thus far, a number of researches have been successfully conducted using cell internalizing aptamers to deliver siRNAs into cancer cells. siRNAs can be conjugated with the internalizing aptamers covalently by using one strand of the siRNA, either sense or antisense, directly or indirectly by using a short linker RNA sequence; they can also be conjugated with the aptamers non-covalently by exploiting streptavidin-biotin interaction or through a short region of complementary base pairing called “sticky bridge”. Chu et al. [122] constructed an aptamer–streptavidin–siRNA conjugate (non-covalent linkage), in which two biotinylated aptamers targeting PSMA and two biotinylated siRNAs targeting lamin A/C mRNA were bound to a tetrameric streptavidin molecule. The assemblies were endocytosed by the PSMA-expressing LNCaP (a human prostate adenocarcinoma cell line) cells and efficiently inhibited the expression of the lamin A/C gene. 

McNamara et al. [123] generated covalently linked aptamer–siRNA chimeric molecules to specifically deliver therapeutic siRNAs targeting PLK1 and BCL2 (two survival genes that are overexpressed in most human cancers) mRNAs into prostate cancer cells expressing the surface receptor PSMA. After internalizing the cells, the constructs were directed to the RNAi pathway, cleaved by Dicer, and silenced their cognate mRNA molecules which resulted in cell death. The aptamer–siRNA chimera was constructed by covalently linking the 3′ end of a PSMA-binding aptamer with the 5′ end of the sense strand of a gene-silencing siRNA, which was achieved by co-transcribing the aptamer and siRNA sense sequence; the antisense strand was then hybridized to the sense strand. 

HER2 has been employed as a target to deliver siRNAs into HER2-positive breast cancer cells. Thiel et al. [124] selected RNA aptamers that are specific to HER2 and can be internalized upon binding. The RNA aptamers were covalently linked to siRNAs targeting Bcl2, an apoptosis suppressing gene. The HER2 aptamer–Bcl2 siRNA conjugates were specifically internalized into HER2-positive cells and silenced the expression of Bcl2 gene. In addition, the silence of the Bcl-2 gene resulted in the sensitization of these cells to the chemotherapeutic drug cisplatin. HER2 aptamers have also been used to deliver EGFR (epidermal growth factor receptor) siRNAs into HER2-positive cancer cells [125,126].

The same or similar conjugating methods as mentioned above have been used by a number of other investigations to construct aptamer–siRNA chimeric RNAs to deliver siRNAs into cancer cells [127,128,129,130,131,132,133,134,135].

#### 5.2.3. Delivery of Nanocarriers

Using aptamers to deliver nanoparticles loaded with drugs is a promising approach to drug delivery. Aptamers are only a few nanometers in diameter, and with this small size, they do not increase much to the overall size of the nanocarriers. Thus, the resulting nanoparticle–aptamer conjugates can relatively easily penetrate the microvasculature and the interstitial tissue of tumors [136]. In addition, aptamers are chemically synthesized and this makes it relatively easy to attach functional groups to their ends and link them covalently to nanoparticles [137].

The first example of nanoparticle–aptamer conjugates used the A10 aptamers targeting PSMA [138]. The authors generated the nanoparticles by synthesizing poly(lactic acid)-block-polyethylene glycol copolymer with a terminal carboxylic acid functional group; they then encapsulated the anticancer drug dextran within these nanoparticles. Next, they conjugated the A10 RNA aptamers that target PSMA to the nanoparticles. The authors found that these nanoparticle–aptamer conjugates could efficiently target the PSMA-positive prostate LNCaP cells and be internalized while the boosted uptake of these conjugates was not observed in cells that do not express PSMA. This nanoparticle–aptamer construction was later assessed in vivo and was demonstrated to be able to reduce the tumor size of PSMA-positive prostate carcinomas effectively [139]. 

Several studies used the HER2 molecule as a target to deliver nanoparticles to cancer cells for therapeutic purposes. One study conjugated HER2 aptamers with dextran-coated ferric oxide nanoparticles and applied them to induce magnetic hyperthermia in human adenocarcinoma SK-BR3 cells that overexpress HER2 receptors [140]. The HB5 aptamer was used to functionalize mesoporous silica–carbon nanoparticles loaded with DOX. The construction (MSCN–PEG–HB5/DOX) was expected to exert a chemo-photothermal combined therapeutic effect on HER2-positive breast cancer cells. The researchers found that the MSCN–PEG–HB5/DOX assembling exhibited significantly higher cellular uptake in the HER2-positive SK-BR-3 breast cancer cell line but not in the normal breast epithelial cell line. The uptake was based on the receptor-mediated mechanism that was energy-dependent. Cytotoxicity experiments showed that the chemo-photothermal combined therapy using the MSCN–PEG–HB5/DOX complex led to a much higher cytotoxic effect than either chemotherapy or photothermal therapy alone [109]. Another study conjugated an HER2 aptamer to curcumin-loaded human serum albumin nanoparticle that could be taken up by HER2-overexpressing SK-BR-3 cells, having the effect of remarkable cytotoxicity in these cells [141]. 

Other nanoparticle–aptamer conjugates that have been reported thus far include: conjugating the anti-CD44 aptamer to liposomes loaded with siRNA for gene silencing in CD44-expressing tumor cells in vivo [142], mucin1 aptamer-conjugated chitosan nanoparticles loaded with docetaxel and cMET siRNA that was delivered into mucin1 positive SKBR3 breast cancer cells [143], LA1 aptamer-conjugated grapefruit-derived nanovectors harboring Dox and P-glycoprotein siRNA that could be internalized into multidrug resistance LoVo colon cancer cells [144], self-assembled DNA nanostructures (DNA nanoprisms) decorated with therapeutic siRNAs targeting the GTPase Rab26 and MUC-1 aptamers that target non-small cell lung cancer [145], and paclitaxel-encapsulated PEGylated PLGA nanoparticles that were surface-functionalized with the heparanase aptamers targeting triple-negative breast cancers [146].

### 5.3. Immunomodulation

Up to the present time, a number of aptamers have been developed that are capable of modulating immune responses against cancer cells. These aptamers were used either as antagonists of immune checkpoints or as agonists of immuno-stimulatory receptors [147]. 

#### 5.3.1. Immune Checkpoint Antagonists

**Figure 3:**
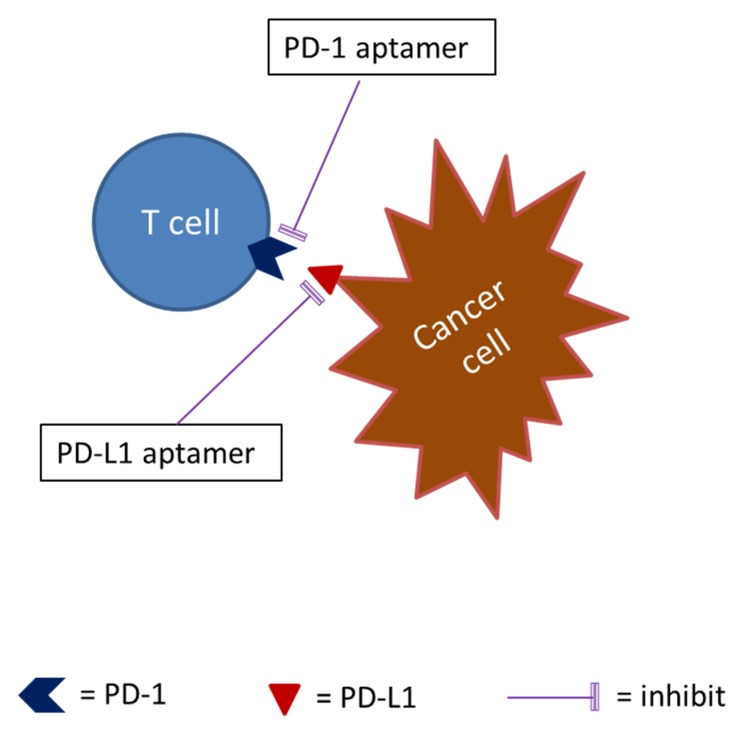
Aptamers as immune checkpoint inhibitors. Note: The binding of programmed death-ligand 1 (PD-L1), the immune checkpoint receptor on T cells, with PD-L1, the ligand on cancer cells, will transduce an inhibitory signal to the T cell. Either programmed death 1 (PD-1) aptamer or PD-L1 aptamer can block the binding.

The recently developed immune checkpoint inhibitors of such programmed death 1 (PD-1) inhibitors and programmed death-ligand 1 (PD-L1) inhibitors have revolutionized cancer immunotherapy [148]. Nevertheless, cancer treatment with PD-1 inhibitors and PD-L1 inhibitors benefited only a subset of patients or some type of cancers, the majority of cancer patients did not show complete responses, and adverse reactions have been observed [148]. The search for new checkpoint targets and new checkpoint inhibitors is still necessary (see Figure 3).

Currently, the most prevalent immune checkpoint receptors that have been used to manipulate the immune system for cancer immunotherapy are PD1 and cytotoxic T-lymphocyte-associated protein 4 (CTLA-4); both are T-cell surface receptors. However, there are many other immune checkpoints that can be employed to modulate the immune system to enhance cancer immunity. These include T-cell immunoglobulin and mucin-domain containing-3 (TIM3), lymphocyte activating 3 (Lag3), B- and T-lymphocyte attenuator (BTLA), adenosine A2A receptor (A2AR), as well as a number of intracellular immune checkpoints such as forkhead box protein P3 (Foxp3), signal transducer and activator of transcription 3 (STAT3), casitas B-lineage lymphoma-b (Cbl-b), early growth response proteins-2 (EGR-2), Src homology region 2 domain-containing phosphatase-1 (SHP1), Src Homology region 2 domain-containing phosphatase-2 (SHP2), etc. Aptamers have been produced for most of these immune checkpoints up to now. 

The first report using aptamers to manipulate immune responses against cancer by antagonizing the immune checkpoint receptor CTLA-4 was the development of RNA aptamers that could bind CTLA-4 with high affinity and specificity and inhibited CTLA-4 function in vitro and enhanced tumor immunity in mice; tetrameric assemblies of the aptamers could improve their bioactivity significantly both in vitro and in vivo [149]. 

DNA aptamers that bind specifically to the extracellular domain of PD-1 have been in development and blocked murine PD-1 and PD-L1 interaction; one of the aptamers functionally inhibited PD-L1 mediated suppression of interleukin-2 secretion in primary murine T cells. Its PEGylated form suppressed the growth of PD-L1 positive murine colon carcinoma cells in vivo with a potency comparable to the anti-PD-1 antibody [112]. Lai et al. [113] reported that a DNA aptamer against human PD-L1 blocked the binding between human PD-1 and PD-L1. They showed that the aptamer promoted lymphocyte proliferation in vitro and suppressed tumor growth in vivo with minimum liver and renal toxicity. Tumors treated with the aptamer showed elevated infiltration of CD4+ and CD8+ T cells and raised levels of IL-2, tumor necrosis factor-α (TNFα), interferon-γ (IFNγ), and C-X-C chemokine ligands CXCL9 and CXCL10. The CD8+ T cells in the treated tumors displayed higher CXCR3 expression than control. 

TIM3 is an immune checkpoint receptor expressed on multiple immune cells that has demonstrated several unique properties and is one of the most promising immune checkpoint targets other than CTLA-4 and PD-1 [150]. TIM3 protein is a member of the TIM family that includes TIM-1, TIM-3, and TIM-4. They are type-I cell-surface glycoproteins comprising extracellular signal peptides and IgV domains, mucin-like transmembrane domains, and an intracellular cytoplasmic tail.

TIM3 was first identified as a molecule selectively expressed on CD4 + IFNγ–producing Th1 and CD8 + cytotoxic Tc1 cells that function to limit the magnitude and duration of Th1 and Tc1 responses [151]. Later studies have shown that TIM3 is expressed on numerous immune cells; in addition to Th1 and Tc1 cells, there are Tregs (regulatory T cells), Th17 cells, TILs (tumor-infiltrating lymphocytes), and innate immune cells. The observations that TIM3 marks dysfunctional phenotype of CD8+ T cells in both experimental models and cancer patients and that TIM3 + Tregs are found solely in tumor tissue have made TIM3 pathway a very suitable target for anticancer immunotherapy based on immune checkpoint blockade [151]. 

Aptamers have been developed to antagonize TIM3, one of which, TIM3Apt, has been shown to bind to the extracellular motives of TIM3 with high affinity and specificity and its monomeric form displayed a potent antagonist capacity in TIM3-positive lymphocytes. Combinational treatment with TIM3Apt and PD-L1 inhibitor showed a synergistic effect in colon carcinoma-bearing mice [152]. A trimeric form of TIM3 aptamer blocked the interaction of TIM3 with Galectin-9, boosted cell proliferation and cytokine secretion, and reduced cell death in vitro. In tumor-bearing mice, the trimeric aptamer delayed tumor growth and enhanced the survival of the tumor-bearing animals used either independently or in combination with a PD-1 inhibitor. In particular, the trimeric aptamer displayed better activity than the currently commercially available monoclonal antibody, RMT3-23, both in vitro and in vivo [153].

#### 5.3.2. Immune Stimulation Agonists

The anti-tumor immunity can also be boosted by providing artificial stimulatory or costimulatory signals to immune cells. Most costimulatory receptors in lymphocytes exert their function through crosslinking their cytoplasmic domains, thus the desired immuno-stimulatory agents need to be able to bring together the receptors to initiate the activation signaling. Antibodies are well suited to crosslinking receptors because of their bivalent antigen-binding construction. To meet that requirement, the first immuno-stimulatory aptamer was designed as a dimer [154]. The aptamer was developed as a murine 4-1BB costimulatory receptor agonist. 4-1BB, also known as CD137 and tumor necrosis factor receptor superfamily member 9 (TNFRSF9), is a type 2 transmembrane glycoprotein receptor belonging to the TNF superfamily. 4-1BB is an important costimulatory receptor expressed on activated T Lymphocytes and supports the survival and expansion of activated T lymphocytes. 

The 4-1BB agonistic aptamer could bind 4-1BB expressed on the surface of activated murine T lymphocytes and stimulate the activation of the T cells in vitro and mediate tumor rejection in mice [154]. To reduce the non-specific immuno-cytotoxic damage to normal tissue or cells, several investigations constructed bi-specific aptamers that bind to 4-1BB as well as to a tumor-specific target either on tumor cells or in tumor stroma. Pastor and others [155,156] conjugated an agonistic 4-1BB aptamer to a PSMA aptamer, which resulted in enhanced antitumor immunity in subcutaneously implanted tumors and lung metastasis in mice. Berezhnoy et al. [157,158] conjugated an agonistic 4-1BB aptamer to an siRNA targeting mTOR complex 1 and found that systemic administration of the conjugates to mice downregulated mTOR complex 1 activity in CD8 + T cells and led to a potent memory response that showed cytotoxic effectors and boosted vaccine-induced immunity in tumor-bearing mice. Schrand et al. [159,160] conjugated an agonistic 4-1BB aptamer to an aptamer that binds to VEGF that is widely expressed in tumor stroma; systemic administration of the conjugates in preclinical murine tumor models exhibited potent antitumor immunity against multiple unrelated tumors and an enhanced therapeutic index. 4-1BB aptamer has been used to conjugate to an siRNA against IL-2Rα whose signaling negatively regulates activated CD8+ T cells. Systemic administration of 4-1BB aptamer-IL-2Rα siRNA conjugates downregulated IL-2Rα mRNA in 4-1BB-positive CD8+ T cells and promoted their differentiation into memory cells [161]. 4-1BB aptamer has also been used in conjugation with an siRNA against Smad4 in the TGFβ signaling pathway, the signaling of which mediates immune suppression at the tumor microenvironment. The 4-1BB aptamer-Smad4 siRNA conjugates rendered T cell resistant to TGFβ inhibition, and systemic administration of the conjugates to tumor-bearing mice boosted irradiation- and vaccine-induced antitumor immunity [162].

Other aptamers generated as agonists to activate costimulatory receptors include the dimeric aptamer against murine OX40 [163], the dimeric aptamers against CD28 [164], the dimeric aptamers against CD40 [165], the aptamer against ICOS [166], and the aptamer against human OX40 [167]. These aptamers were used independently or in combination with immune checkpoint inhibitors.

## 6. Cancer Therapy Aptamers in Clinical Trials

In addition to Macugen, which was approved by the FDA more than 10 years ago for the treatment of ADM, there have been a number of aptamers that have entered clinical trials. Table 2 shows the selected aptamers that are under clinical trials or in active laboratory investigation for cancer therapy.

## 7. Conclusions

Aptamers are single-stranded RNA or DNA oligonucleotides generated with SELEX technology. Aptamers can form numerous specified three-dimensional structures and thus can bind diverse target molecules with high affinity and specificity. Like mAbs, aptamers have promising potential applications in clinical diagnosis and therapy particularly in cancer treatment, but aptamers have advantages over mAbs in therapeutic uses mainly because they have little or no immunogenicity, which means the feasibility of repeat prescriptions and fewer side effects. Researches on aptamers are still in the early stage, and a deeper understanding of aptamer–target interactions and pharmacokinetics is still needed to prepare for better clinical use or marketing. So far, although a number of aptamers have been developed and some of them have entered clinical trials, only one aptamer has been approved by the FDA for clinical use. The progress towards effective therapies tends to be slow, but it never ceases and the best results are yet to come. With regard to cancer treatment, in addition to their advantage of little or no immunogenicity, aptamers are relatively easy to penetrate solid tumor tissue because of their smaller molecular weight and are comparatively less costly than mAbs since they are produced in vitro. Therefore, more efforts should be devoted to the discovery of new anticancer aptamers, to the in-depth characterization of the current aptamers, and to speeding up the process for the entry of existing anticancer aptamers into clinical trials.

## Figures and Tables

**Table 1:** A comparison between aptamers and monoclonal antibodies (mAbs).

Criteria	Aptamers	mAbs
Chemical composition	Nucleic acid (DNA or RNA)	Protein
Molecular weight	10–50 kDa	140–700 kDa
Animal immunization for preparation?	No	Yes (except for genetically engineered Ab)
In vitro preparation	Yes	No
Targets [21,22,23]	Multiple, including cells, viruses, proteins, peptide, polysaccharides, nucleic acids, nucleotides, amino acids, other small organic molecules and inorganic molecules, etc.	Proteins mainly, but include cells, viruses, polysaccharides, and nucleic acids
Specificity to target	Yes	Yes
Binding affinity	Nano-molar~pico-molar, maybe femto-molar	Nano-molar~pico-molar, maybe femto-molar
Molecular forces involved in target binding	Electrostatic forces, hydrogen bonds, hydrophobic interactions, and van der Waals forces	Electrostatic forces, hydrogen bonds, hydrophobic interactions, and van der Waals forces
Stability	Stable at 80 °C	denatured at 80 °C
Reannealing if denatured	Yes	No
batch-to-batch variations	Low	High
Shelf life	Long	Short
Cost	Lower	Higher
In vivo half-life [24] (clearance rate)	Short (~20 min)	Long (~one month)
Immunogenicity (causing allergy)	No	Yes (unless humanized)
Internalization	Higher possibility	Difficult
Diagnostic usage	Yes	Yes
Therapeutic usage	Yes	Yes, but may cause allergy if not humanized

**Table 2:** Selected aptamers under clinical trials or active laboratory investigation for cancer therapy.

Aptamer	Selection	DNA/RNA	Kd	Target	INT	Function	Application/Mechanism	Status	References
Pegaptanib sodium (Macugen)	SELEX, 10 rounds	RNA (28 nt)	50 pM	VEGF_165_	No	Antagonism	1. Age-related macular degeneration (AMD). 2. Potential therapeutic application for solid cancers with extensive angiogenesis.	Approved by FDA for treatment of AMD	[84,85,86,87]
AS1411	Designed and chemically synthesized	DNA (26 nt), guanosine rich quartets	55 nM	Nucleolin	Yes	Internalization or delivery	1. Binding cell-surface nucleolin and internalization, leading to DNA replication inhibition. 2. Drug delivery.	Phase II clinical trial	[18,88,89,90]
NOX-A12	Spiegelmer * technology	L-RNA (45 nt)	200 pM	CXCL12	No	Antagonism	Disrupting the homing and the accumulation of CLL cells in the bone marrow, sensitizing these cells to cytotoxic drugs.	Phase II clinical trial	[54,81,83]
AX102	SELEX, 12 rounds	DNA (34 nt)	100 pM	PDGF-B	No	Antagonism	1. Inhibition of tumor angiogenesis. 2. Promotion of tumor blood vessel efficiency, resulting in increased anticancer drug delivery.	Pre-clinical	[67,69,70,71]
xPSM-A10 (A10)	SELEX, 6 rounds	RNA (72 nt)	1.5 nM	PSMA	Yes	Internalization and delivery	# Delivery of (1) chemotherapeutics, (2) therapeutic RNAs, and (3) nanoparticles to PSMA-positive prostate cancer cells.	Pre-clinical	[91,92,93,94,95,96,97,98,99,100,101,102,103,104,105,106,107]
HB5	SELEX, multiple rounds	DNA (86)	18.9 nM	HER2	Yes	Internalization and delivery	Delivery of (1) chemotherapeutics, (2) nanoparticles to HER2-positive breast cancer cells.	Pre-clinical	[108,109,110]
HeA2_3	Whole-cell SELEX	DNA	6.2 nM	HER2	Yes	Internalization	Binding with high specificity to HER2- positive cells and tumor tissue and great potential for the treatment of HER2- overexpressing cancers.	Pre-clinical	[111]
MP7	SELEX, 5 rounds	DNA	167 nM	Murine PD-1	No	Antagonism	Blocking murine PD-1 and PD-L1 interaction so as to restore T cell function.	Pre-clinical	[112]
aptPD-L1	SELEX, 8 rounds	DNA	4.7 nM	Human PD-L1	No	Antagonism	Blocking the binding between human PD-1 and PD-L1 so as to restore T cell function.	Pre-clinical	[113]

Abbreviations: INT (internalization); AMD (age-related macular degeneration); CLL (chronic lymphocytic leukemia); HER2 (human epidermal growth factor receptor 2); CXCL12 (C-X-C Chemokine Ligand 12); Kd (dissociation constant), PD-1 (programmed death 1); PD-L1 (programmed death-ligand 1); PDGF-B (platelet-derived growth factor receptor-B); PSMA (prostate-specific membrane antigen); SELEX (systematic evolution of ligands by exponential enrichment); VEGF_165_ (165-amino-acid isoform of vascular endothelial growth factor). * See main text for the explanation of Spiegelmer. # Including a truncated version of A10 (A10-3.2).

## Abbreviations

A2AR	adenosine A2A receptor
AMD	age-related macular degeneration
BTLA	B- and T-lymphocyte attenuator
Cbl-b	casitas B-lineage lymphoma-b
CTLA-4	cytotoxic T-lymphocyte-associated protein 4
CXCL	C-X-C chemokine ligand
CXCR	C-X-C chemokine receptor
Dox	doxorubicin
EGFR	epidermal growth factor receptor
EGR-2	early growth response proteins-2
Foxp3	forkhead box protein P3
HER2	human epidermal growth factor receptor 2
IFNγ	interferon-γ
Lag3	lymphocyte activating 3
mAb	monoclonal antibody
PD-1	programmed death 1
PD-L1	programmed death-ligand 1
PDGF	platelet-derived growth factor
PDGFR	platelet-derived growth factor receptor
PSMA	prostate-specific membrane antigen
SELEX	systematic evolution of ligands by exponential enrichment
SHP1	Src homology region 2 domain-containing phosphatase-1
STAT3	signal transducer and activator of transcription 3
TALL	T-cell acute lymphoblastic leukemia
TIM3	mucin-domain-containing-3
TNFα	tumor necrosis factor-α
Treg	regulatory T cell
VEGF	vascular endothelial growth factor
